# Structural and Functional Disruption of Thiopurine S‑Methyltransferase by the A80P Variant: A Simulation and Genotyping Study

**DOI:** 10.1021/acsomega.5c08089

**Published:** 2026-06-17

**Authors:** Kaishiv Joshi, Rahul Kumar, Rakesh Kumar, Satbir Kaur, Sebastian Kmiecik

**Affiliations:** † Department of Human Genetics, 29766 Punjabi University, Patiala 147002, India; ‡ Dr. B.R.A. Institute Rotary Cancer Hospital, 28730 All India Institute of Medical Sciences, New Delhi 110029, India; § Biological and Chemical Research Center, Faculty of Chemistry, 49605 University of Warsaw, Warsaw 02-089, Poland

## Abstract

Thiopurine S-methyltransferase (TPMT) is a cytosolic enzyme involved in the metabolism of thiopurine drugs such as 6-mercaptopurine, 6-thioguanine, and azathioprine. Genetic polymorphisms in TPMT can reduce enzyme activity and increase the risk of adverse drug reactions. One such variant, TPMT2 (A80P), has been reported to impair TPMT function. In this study, we used molecular dynamics simulations, molecular docking, and umbrella sampling to investigate how the A80P mutation affects TPMT’s structure and its interaction with the cofactor S-adenosylmethionine (SAM). Our results indicate that the mutation increases structural flexibility, reduces stability, and weakens SAM binding, suggesting compromised enzymatic function. Although TPMT2 is not directly linked to acute myeloid leukemia (AML), identifying its prevalence in AML patients has clinical value due to the potential use of thiopurines in treatment. We screened 50 AML patients and 50 healthy controls from northern India for the TPMT2 variant using PCR-based genotyping. The variant was absent in all individuals tested. These findings support the importance of population-specific pharmacogenetic screening. Tailoring genetic tests to local variant frequencies can improve cost-effectiveness, reduce unnecessary testing, and contribute to safer, more personalized cancer therapy.

## Graphical abstract

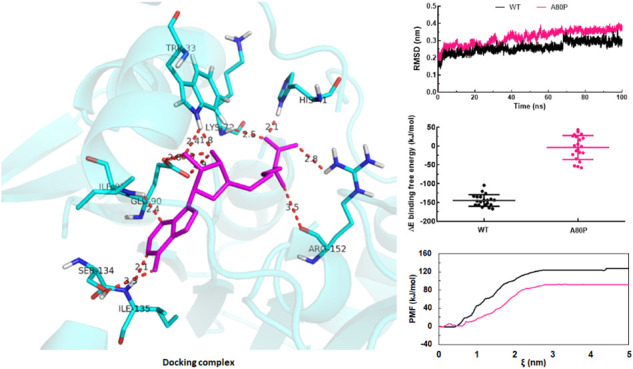

## Introduction

Drug metabolism is the biochemical process by which drugs are converted into inactive, nontoxic compounds or into pharmacologically active metabolites and is a critical aspect of clinical pharmacology.^1,2^ This transformation occurs primarily through enzymatic reactions in the liver and typically involves two main phases. Phase I reactions, catalyzed mainly by cytochrome P450 (CYP450) enzymes, involve the introduction or unmasking of polar groups, while Phase II reactions involve conjugation with endogenous molecules, facilitating excretion.^3^ Enzymes such as N-acetyltransferases, glutathione S-transferases, sulfotransferases, and methyltransferases, including thiopurine S-methyltransferase (TPMT), participate in Phase II metabolism.

The goal of these metabolic processes is to convert lipophilic drug compounds into hydrophilic forms that can be efficiently eliminated from the body with minimal toxicity.^4^ However, interindividual variability in drug metabolism is a well-recognized phenomenon and contributes to a variable drug response (VDR). Some patients exhibit slow metabolism, while others metabolize drugs rapidly, leading to therapeutic failure or toxicity.^5^ These differences arise from a combination of genetic factors, particularly single nucleotide polymorphisms (SNPs), and nongenetic factors such as age, diet, or drug interactions. Among these, genetic polymorphisms are particularly critical, as they vary between individuals and populations.^5^

TPMT is a key cytosolic methyltransferase that catalyzes the S-methylation of aromatic and heterocyclic sulfhydryl compounds, including thiopurine drugs such as 6-mercaptopurine (6-MP), 6-thioguanine (6-TG), and azathioprine.^6^ The enzyme exhibits genetic polymorphism, which can significantly affect its activity. It has been reported that approximately 11% of individuals have reduced TPMT activity, and about 0.3% exhibit complete TPMT deficiency.^7^ To date, over 30 TPMT variants have been identified, most of which involve nonsynonymous SNPs. Among them, **TPMT2 (238G > C), 3A (460G > A and 719A > G), 3B (460G > A), and 3C (719A > G) are the most common and account for 80–95% of intermediate or deficient TPMT activity.^8^

Patients with TPMT deficiency are unable to properly metabolize standard doses of thiopurines, placing them at high risk of severe or even fatal hematological toxicity.^9^ Because thiopurines are used in the treatment of various leukemias, inflammatory diseases, and organ transplantation, TPMT genotyping has become a key component of pharmacogenetic testing. Notably, TPMT2 is more frequently observed in Caucasian and African-American populations, while it appears to be rare or absent in Asian populations, including those in Southwest Asia.^10–12^

The TPMT2 variant involves a G-to-C transition at nucleotide 238, leading to the substitution of alanine with proline at position 80 (A80P) in the TPMT protein. This substitution is located in an α-helical region, potentially disrupting the local secondary structure and protein stability. Previous short-timescale molecular dynamics simulations have indicated that A80P may destabilize the TPMT structure.^13^ While some studies have examined TPMT’s interaction with S-adenosylhomocysteine (SAH) or 6-MP,^14^ no study to date has focused on how TPMT mutations influence the binding of its essential cofactor, S-adenosylmethionine (SAM), which donates the methyl group during catalysis.^15^ SAM is also known to stabilize the TPMT protein, further underlining the importance of studying its interaction with TPMT variants.

In the present study, we investigated the structural and functional consequences of the TPMT2 (A80P) variant using classical molecular dynamics and enhanced sampling methods. We specifically focused on protein conformational changes and the SAM binding affinity. Furthermore, to understand the clinical relevance of this variant in a local context, we performed genotypic screening for TPMT2 in acute myeloid leukemia (AML) patients from the Punjab region (southwest Asia) using allele-specific PCR. Although TPMT is not involved in the development of AML, its activity may still be clinically relevant to these patients. Thiopurine drugs could be used as part of supportive or adjunctive therapy, and in such cases, TPMT deficiency may increase the risk of severe drug-related toxicity.

## Computational Methods

### Structure Modeling and Molecular Docking

The tertiary structure of thiopurine S-methyltransferase (TPMT) is available in the Protein Data Bank (PDB ID: 2BZG) at a resolution of 1.89 Å.^16,17^ Missing residues in the crystal structure were modeled using Modeler 10.5.^18^ The resulting full-length structure was designated as the wild type (WT). The A80P variant was generated by substituting alanine with proline at position 80, and its tertiary structure was similarly modeled using Modeler. Both WT and A80P models were energy-minimized, and their stereochemical quality was subsequently assessed.^19,20^

The refined and optimized WT and A80P structures (obtained from MD simulation snapshots) were then used as inputs for molecular docking. The 2D structure of the S-adenosyl methionine (SAM) ligand (PubChem ID: 34755) was retrieved from the PubChem database, and 3D coordinates with explicit hydrogen atoms were generated using OpenBabel.^21^ The receptor and ligand were prepared using AutoDock Tools by adding polar hydrogens, merging nonpolar hydrogens on the receptor, and assigning Gasteiger charges to the ligand, respectively.^22^

A grid box of dimensions 24 × 24 × 24 Å was defined, centered at *x* = 18.823, *y* = 11.676, *z* = 14.262, around the ligand-binding site identified in the PDB structure. Docking protocol validation was performed by redocking the native ligand using the same grid parameters, and the resulting pose was compared to the crystallographic one.

Final docking simulations were carried out using AutoDock Vina v1.2.x.^23^ The accuracy of docking results was evaluated by calculating the root-mean-square deviation (RMSD) between the predicted and experimental poses. The top-scoring docked complexes were selected for further refinement via MD simulation, and their binding energies were subsequently reevaluated.

### Molecular Dynamics Simulation and Principal Component Analysis

The structural dynamics of apo proteins (WT and A80P) and protein–ligand complexes (WT-SAM and A80P-SAM) were assessed using all-atom molecular dynamics (MD) simulations with the GROMACS 2021.1 suite, as described previously.^24^ Protein and ligand topologies were generated using GROMACS and the SwissParam server, respectively, with the CHARMM27 force field applied.^25,26^ All four systems were solvated in a triclinic water box with a buffer distance of 1.2 nm between the protein surface and the box boundary. Each system was neutralized using sodium and chloride counterions, followed by energy minimization using the steepest descent method.

Subsequently, two equilibration steps were performed: 100 ps in the NVT ensemble and 500 ps in the NPT ensemble. The system temperature and pressure were maintained at 310 K and 1 bar using the modified Berendsen thermostat and the Parrinello–Rahman barostat, respectively.^27^ The Berendsen thermostat is used during equilibration, and the Nosé–Hoover thermostat is used during production MD. Electrostatic interactions were calculated using the Particle Mesh Ewald (PME) method.^28^ A 100 ns production run was performed for each system in triplicate, with a 2 fs time step and trajectory data saved every 2 ps. The initial protein structure was used as a reference to calculate the RMSD, and trajectory analyses were conducted using standard GROMACS tools.

Principal component analysis (PCA) was applied to the protein backbone atoms to investigate collective motions in each system. A covariance matrix was constructed and diagonalized to obtain eigenvectors (principal components, PCs) and their corresponding eigenvalues.^29^ The top few PCs were selected based on their contribution to the total motion, and their cosine contents were analyzed to exclude random noise.^30^ The dominant PCs, representing global protein motions, were plotted and analyzed to assess the conformational differences between systems. PCA was performed using the gmx covar, gmx anaeig, and gmx analyze modules of GROMACS.

### Binding Free Energy Calculation and Umbrella Sampling Simulation

Binding free energies for the WT and A80P complexes were computed from the last 20 ns of the equilibrated MD trajectories. van der Waals, electrostatic, polar solvation, and solvent-accessible surface area (SASA) energy components were combined to estimate the overall binding free energy. Calculations were performed using the Poisson–Boltzmann surface area (PBSA) method via the g_mmpbsa script.^31^

To explore ligand unbinding and conformational transitions, steered molecular dynamics (SMD) and umbrella sampling (US) simulations were performed.^32^ SMD simulations were initiated from the most stable docked conformations obtained from conventional MD. Both WT and A80P complexes were placed in a simulation box of size 13.1 × 8.6 × 24 Å. The pulling direction was aligned with the longest box axis, and the box size was chosen to fully accommodate the 5 nm displacement without periodic artifacts. The reaction coordinate (RC) was defined as the center-of-mass (COM) distance between the protein and the ligand. COM distances ranging from 0.5 to 5 nm along the *z*-axis, spaced at 0.2 nm intervals, were used to generate 20 sampling windows.

Each umbrella sampling window was subjected to 10 ns of MD simulation. The system’s temperature and pressure were maintained at 310 K and 1 bar using the Nosé–Hoover thermostat and the Parrinello–Rahman barostat, respectively. A harmonic potential with a force constant of 1000 kJ/mol·nm^2^ was applied in each window to guide ligand unbinding. Finally, data from all windows were processed using the Weighted Histogram Analysis Method (WHAM) to calculate the potential of mean force (PMF) profile.^33^

## Experimental Methods

### Subjects

A total of 50 patients diagnosed with acute myeloid leukemia (AML) and 50 age- and sex-matched healthy controls were included in this study. AML patients were recruited following pathological confirmation at the Sandhu Cancer Centre in Ludhiana. The control group consisted of individuals with no personal or family history of cancer, recruited from local blood donation camps. Peripheral blood samples were collected in EDTA-coated vials.

The study was approved by the Institutional Ethical Research Committee (Ethical Approval No. 53), and written informed consent was obtained from all participants, including both patients and controls.

### DNA Isolation and TPMT*2 Genotyping

Genomic DNA was extracted from peripheral blood using an inorganic (salting-out) method and stored at −20 °C until further use. DNA quality and concentration were verified using a spectrophotometer.^34^

Allele-specific PCR (AS-PCR) was used to detect the TPMT2 (238G > C) mutation in exon 5, employing sequence-specific forward and reverse primers (Table 1). Two separate PCR reactions were performed for each subject: one for the wild-type allele and another for the mutant allele, using the P2W and P2C primers, respectively.

**Table 1:** List of Oligonucleotide Primers

Gene	Gene Variation	Sequence (5′-3′)	Length of product (bp)
*TPMT*	238G > C (rs180046)	**P2W** GTATGATTTTATGCAGGTTTG	254 bp
		**P2M** GTATGATTTTATGCAGGTTTC	
		**P2C** TAAATAGGAACCATCGGACAC	

Each PCR reaction was carried out in a final volume of 20 μL, containing 0.5 μM of each primer, 5 μL of genomic DNA, 3 μL of nuclease-free water, and 10 μL of 2× PCR master mix. The PCR conditions included an initial denaturation at 94 °C for 2 min, followed by 35 cycles of 94 °C for 30 s, 53 °C for 30 s, and 72 °C for 30 s, with a final extension at 72 °C for 2 min.

### Statistical Analysis

Statistical analysis was performed by using SPSS software (version 16.0). Chi-square tests were applied to compare genotype frequency distributions between AML patients and healthy controls. A *p*-value of <0.05 was considered statistically significant.

## Results

### Structural and Dynamic Properties of WT and A80P Mutant

We began by examining the tertiary structures of the wild-type (WT) and A80P variant. The WT protein forms a stable α/β fold typical of cytosolic methyltransferases, consisting of eight α-helices (H1–H8) and eight β-strands (S1–S8) (Figure S1).^16,17^ The A80P substitution is located within α-helix 4, near a turn connecting β-sheet 2, suggesting potential destabilization of the local structural integrity.^13^

Here, we ran all-atom MD simulations of 100 ns for both WT and A80P systems. The WT protein reached equilibrium quickly (∼10 ns) and maintained a stable RMSD (∼0.24 ± 0.01 nm), whereas A80P required ∼55 ns to stabilize and exhibited a higher RMSD (∼0.28 ± 0.02 nm) (Figure 1A). Radius of gyration (Rg) analysis showed that A80P was less compact than WT (∼1.82 nm vs ∼1.77 nm) (Figure 1B). RMSF analyses indicated that A80P had greater flexibility, especially at residues ∼120, ∼180, and 220–230 (∼0.6 nm) (Figure 1C). Mapping these onto the structure revealed that residues Glu225, Arg226, Ser229, and Trp230 were particularly mobile (Figure S2). This suggests that the A80P mutation induces local structural disruption and enhances flexibility in regions important for protein stability.

**Figure 1:**
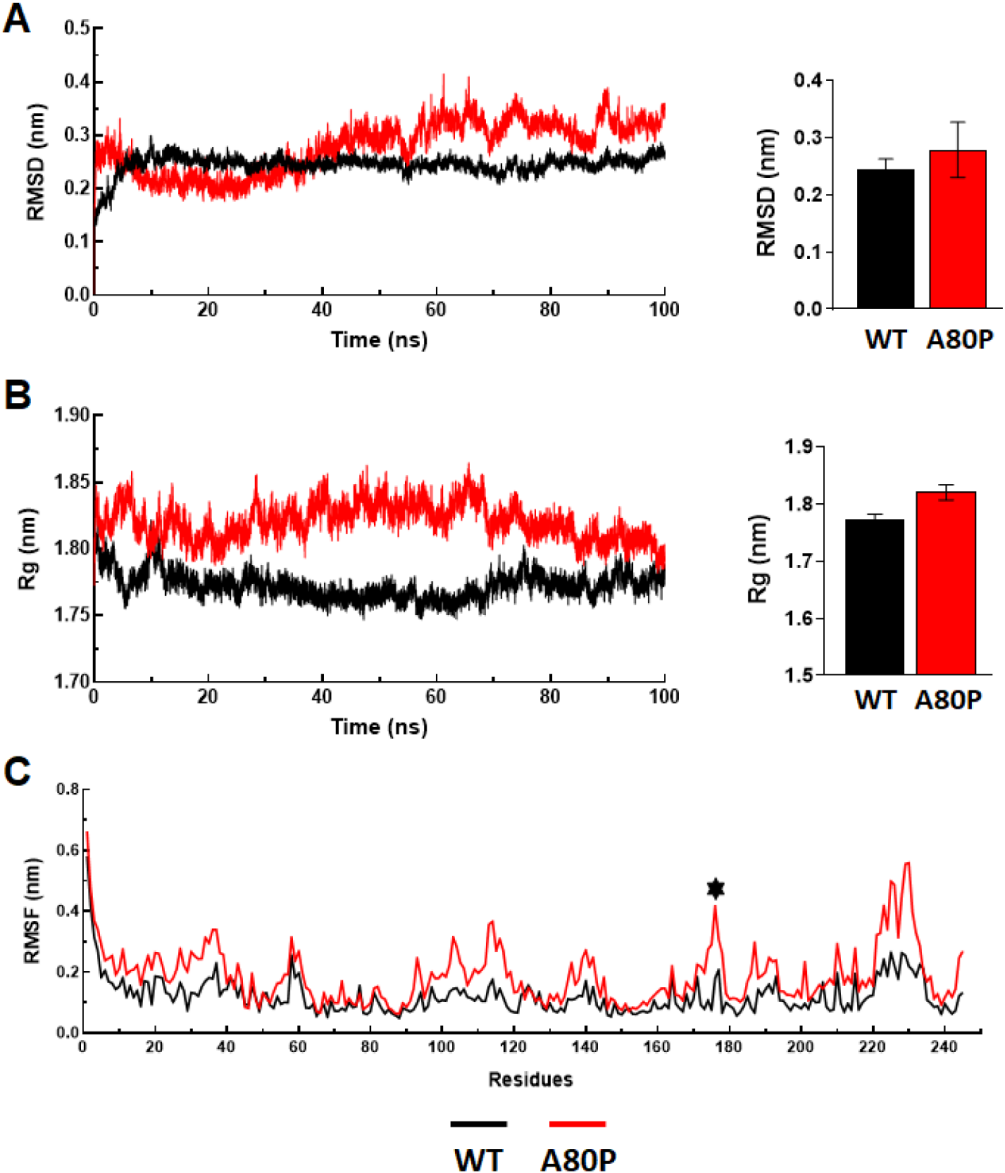
Structural stability and flexibility of WT and A80P proteins during MD simulations. (A) RMSD profiles showing overall structural deviation over time (left) and average RMSD values (right). (B) Radius of gyration (Rg) profiles indicating protein compactness (left) and average Rg (right). (C) RMSF profiles showing residue-level flexibility. WT and A80P are shown in black and red, respectively.

### Changes in Solvent Exposure, Secondary Structure, and Hydrogen Bonding

We next measured features associated with structural integrity based on the analysis of MD simulation trajectories (Figure S3). We calculated the SASA values from three independent simulations. Errors were corresponding to the standard deviations across these replicas. A80P showed consistently higher total solvent-accessible surface area (SASA) (∼133.79 nm^2^) compared to WT (∼124.70 nm^2^), driven by greater hydrophobic exposure (Figure 2A). A residue-level SASA decomposition was performed to determine the individual contributions to total solvent exposure. The results indicated enhanced participation of hydrophobic residues in bonding interactions, with noticeable differences in residue distribution profiles between the WT and A80P systems (Figure S4). Such increases often correlate with the destabilization of the hydrophobic core.^19^

**Figure 2:**
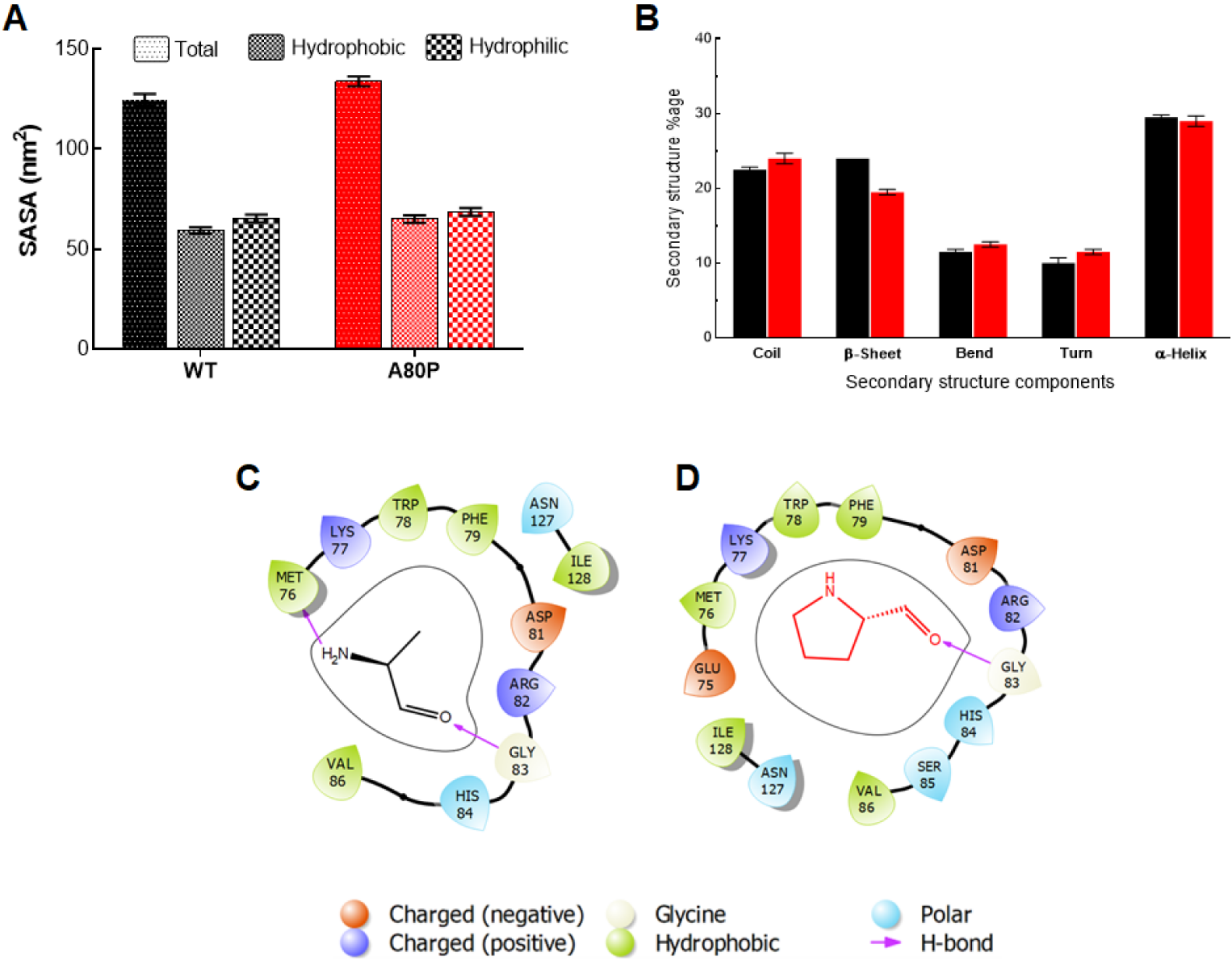
Structural properties and residue interactions. (A) Solvent-accessible surface area (SASA) over time for WT and A80P. (B) Percentage of secondary structure elements (α-helix, β-sheet, coil) throughout simulations. (C, D) Residue–residue interaction 2D maps for WT (C) and mutant (D). 2D interaction plots are generated using the ligand interaction map tool. WT residues are shown in black; mutant residues are shown in red; different interaction types and charges are shown in various colors as indicated in the figure legend.

Secondary structure analysis revealed reduced β-sheet content in A80P (19.5% vs 24% in WT) and increased coil, bend, and turn elements (Figure 2B). Since β-sheets provide structural rigidity,^20^ this shift suggested an overall softening of the protein fold.

Hydrogen bond analysis supported these observations: A80P had fewer intraprotein H-bonds (167 vs 175 in WT) but more protein–solvent H-bonds (544 vs 524), indicating weakened internal cohesion and greater solvent exposure (Figure S3D and E). A80P also exhibited more hydrophobic interactions (13 vs 11), reinforcing the notion of an altered core packing (Figure 2C,D).^19^

### Essential Motions and Conformational Flexibility

To understand how the A80P mutation affects collective motions, we performed principal component analysis (PCA) on the MD trajectories. We constructed the covariance matrices of backbone atomic fluctuations and diagonalized them to extract eigenvectors (principal components) and eigenvalues.^27^ The diagonalized matrices have trace values of 26.46 nm² for WT and 20.42 nm^2^ for A80P MT. Here, we show the first 30 eigenvectors with corresponding eigenvalues, where MT (red) exhibits higher eigenvalues compared to WT (black), indicating that the MT system has greater flexibility and explores a broader conformational space (Figure 3A).

**Figure 3:**
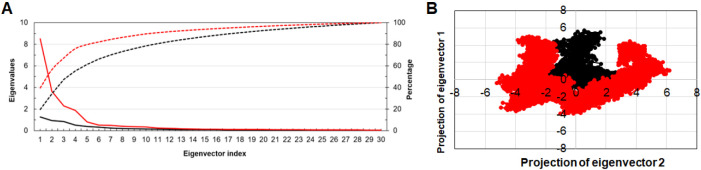
Principal component analysis (PCA) of protein motions. (A) Eigenvalues of the first 30 principal components with their cumulative contribution to overall motion. (B) Projection of the trajectory onto the first two principal components, illustrating the conformational space sampled by WT (black) and A80P (red).

We found that the first two principal components accounted for a larger fraction of overall motion in A80P (∼40–60%) than in WT. When we projected the trajectories onto the first two components, we observed that WT remained confined to a compact conformational space, while A80P explored a broader and more diffuse region (Figure 3A,B). This shift suggests that the A80P mutation expands the accessible conformational landscape, introducing higher flexibility and potential instability in regions critical for cofactor binding and catalysis.^30^

### Ligand Binding, Dynamics, and Energetics

Given that TPMT activity depends on stable SAM binding, we investigated how A80P affects ligand interactions. We began by validating our docking protocol: our predicted SAM pose aligned closely with the experimental structure (RMSD < 0.92 Å) (Figure S5).^23^ Docking scores indicated a slightly stronger predicted binding affinity for the WT (−39.32 kJ/mol) complex compared to the A80P (−38.49 kJ/mol).

Next, we carried out 100 ns MD simulations of SAM-bound complexes. Initial RMSD analysis of the WT complex showed structural stabilization for the first 70 ns, followed by a brief increase of approximately 0.3 nm, after which the system restabilized for the remainder of the simulation (Figure 4A). This transient increase is primarily attributed to local structural movement in the turn connecting two β-sheets, which was not observed in the A80P-SAM complex (Figure S6). In contrast, the A80P mutant complex reached stabilization after approximately 50 ns. Overall, the RMSD analysis indicates that both complexes are stable and exhibit comparable dynamic behavior throughout the simulation period.

**Figure 4:**
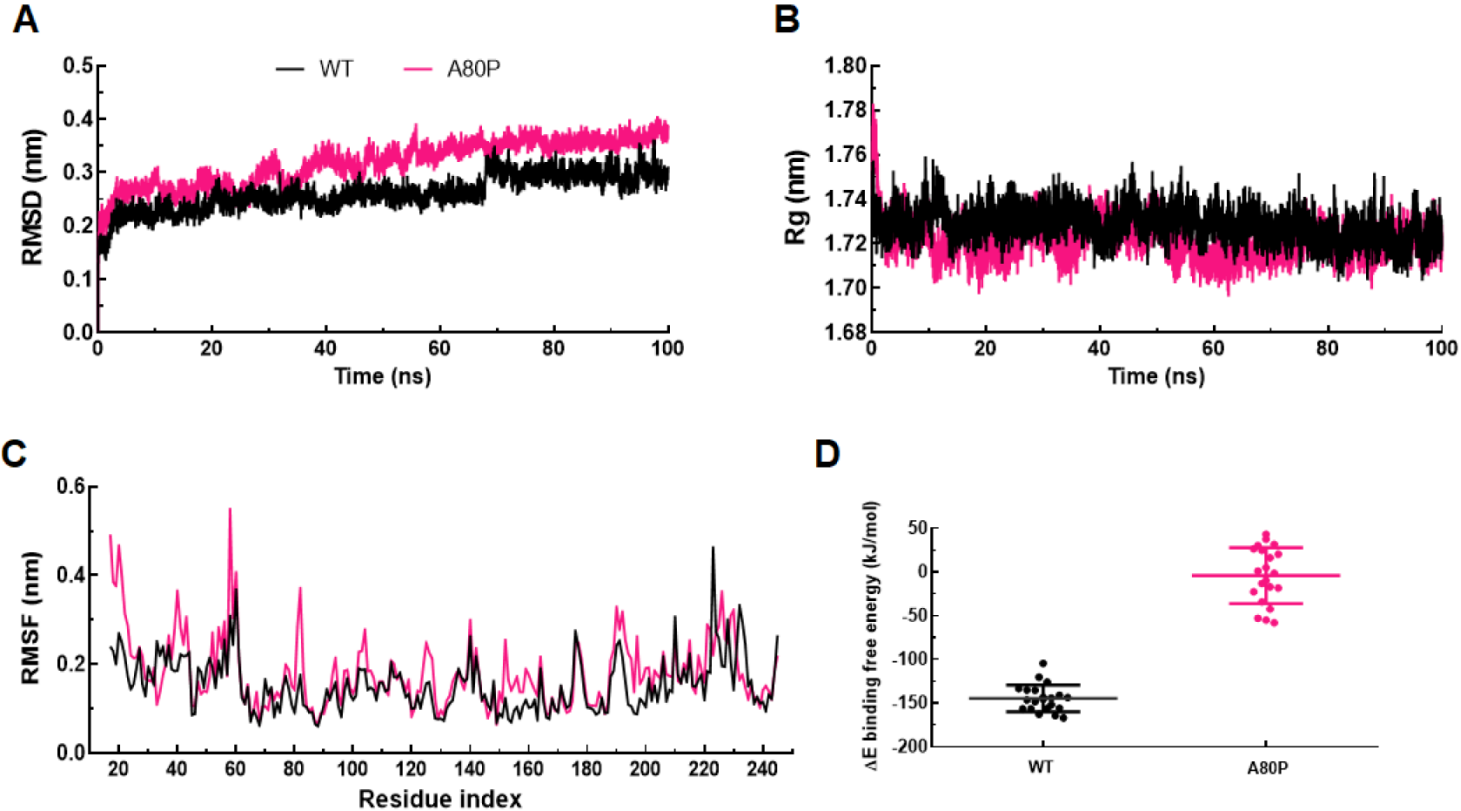
Dynamics and binding analysis of protein-SAM complexes from MD simulations. (A) RMSD profiles, (B) radius of gyration, (C) RMSF profiles, and (D) binding free energy calculations comparing WT (black) and A80P (magenta) complexes.

Both WT and A80P complexes demonstrated comparable Rg profiles, indicating structural stability and compactness during the simulation. Conversely, the apo systems exhibited increased fluctuations, with the A80P mutant showing a notably higher Rg value (Figure 4B). Both WT and A80P reached stable trajectories, but A80P showed elevated local flexibility, particularly around residues 20 and 60 (Figure 4C). Notably, compared to the apo forms of the A80P protein, these residues demonstrated even greater fluctuations in the ligand-bound state, suggesting increased flexibility upon ligand binding. Binding free energy calculations using MM/PBSA demonstrated that WT had a significantly stronger binding affinity (Δ*E* ∼ −144.62 ± 3.2 kJ/mol) compared to A80P (Δ*E* ∼ −4.03 ± 0.12 kJ/mol) (Figure 4D). The lower binding free energy in the WT complex was mainly contributed by van der Waals forces, as more hydrophobic residues were involved in ligand binding. These data imply that A80P disrupts the SAM-binding pocket, impairing interactions critical for catalytic function.

### Umbrella Sampling and Ligand Unbinding

We aimed to further quantify the binding strength and characterize the energetic barriers associated with SAM release. Classical MD simulations often fail to capture rare events, such as ligand unbinding, due to limited simulation time scales.^32^ Therefore, we employed steered MD (SMD) combined with umbrella sampling techniques, which apply external biasing forces to systematically pull the ligand away from the active site, enabling the calculation of the potential of mean force (PMF) profile along a defined reaction coordinate.^33^

We generated 20 sampling windows from SMD trajectories and conducted umbrella sampling simulations, validating the PMF profiles through histogram overlaps and bootstrap analysis (Figure S8). Force profiles during pulling confirmed stronger binding in WT (Figure 5A). The results revealed that WT required significantly more energy to dissociate SAM (>120 kJ/mol) compared to A80P (∼90 kJ/mol) (Figure 5B). Structural snapshots showed that SAM remained engaged in the WT binding pocket for longer, whereas in A80P, it detached more readily (Figure 5C–D). Collectively, these findings indicate that the A80P mutation weakens SAM binding and destabilizes the active site, potentially reducing TPMT enzymatic activity in vivo and increasing the risk of altered thiopurine metabolism and drug-related toxicity.

**Figure 5:**
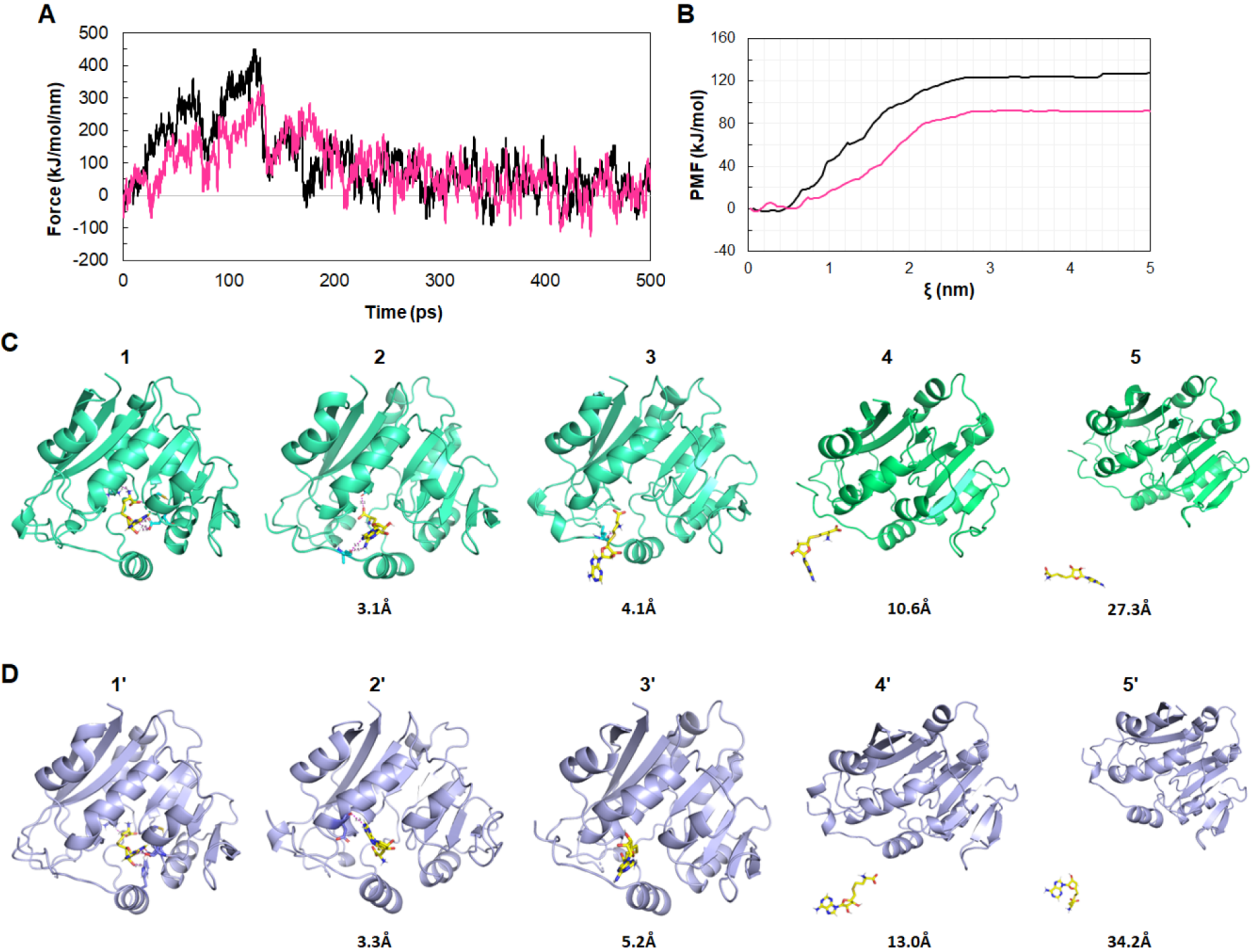
Ligand unbinding simulations using steered MD and umbrella sampling. (A) Force profiles showing the external force required to unbind SAM from WT and A80P proteins. (B) Potential of mean force (PMF) curves quantifying the energy barrier for unbinding. (C) Structural snapshots of 5 time points (1–5) showing the ligand being pulled out from the bound to the unbound state in the WT complex. (D) Structural snapshots of 5 time points (1’-5′) showing the ligand being pulled out from the bound to the unbound state in the A80P complex. The protein is shown as cartoon; ligand as sticks. H-bonds are depicted as magenta dotted lines. The distance between the protein and ligand is measured in angstroms and shown in figure.

### Population Screening of TPMT2 in AML Patients

Lastly, we investigated the prevalence of TPMT2 (A80P) in a clinical cohort. We analyzed 50 AML patient samples and 50 healthy controls using allele-specific PCR.^32^ General characteristics of AML patients and controls are presented in Table 2. Agarose gel electrophoresis images displayed representative AML sample DNA with the expected PCR product size (∼254 bp) (Figure S9). All individuals in our cohort carried only the homozygous wild-type genotype (GG). Neither heterozygous (GC) nor homozygous mutant (CC) genotypes were detected (Figure S6, Table 3). This finding aligns with previous studies indicating that TPMT2 is rare or absent in South Asian populations.^6^ While routine clinical testing for TPMT2 may not be warranted in this region, our structural and computational analyses reinforce the potential functional impact of this variant should it be encountered, highlighting the importance of combining population studies with structural biology approaches in pharmacogenetics research.

**Table 2:** Baseline Characteristics of AML Patients and Controls (*p < 0.05)

Variables		AML (*N* = 50)^a^ *n* (%)	Controls (*N* = 50)^a^ *n* (%)	*p*-value
Mean age ± SD (Range) years		42.45 ± 20.7 (39–60)	38.36 ± 16.02 (35–62)	
Gender	Male	23 (46)	23 (46)	—
	Female	27 (54)	27 (54)	
Dwelling	Urban	21 (42)	28 (56)	0.229 (1.44)
	Rural	29 (58)	22 (44)	
Physical activity	Active	10 (20)	43 (86)	0.0001* (9.04)
	Sedentary	40 (80)	07 (14)	
Dietary habits	Veg	32 (64)	26 (52)	0.233 (1.48)
	Nonveg	18 (36)	24 (48)	
Smoking	Yes	4 (8)	02 (4)	0.677 (0.18)
	No	46 (92)	48 (96)	
Alcohol drinking	Yes	7 (14)	08 (16)	0.788 (0.08)
	No	43 (86)	42 (84)	

a Numbers in brackets denote sample size.

**Table 3:** Genotype Frequency of TPMT2 Polymorphism Among AML Patients and Controls

Genotype		Controls (*N* = 50)^a^ *n* (%)	AML (*N* = 50)^a^ *n* (%)	Chi- square	*p* value
TPMT2 (238G > C) (rs1800462)	GG	50 (100)	50 (100)		
	GC	0 (0)	0 (0)	–	–
	CC	0 (0)	0 (0)	–	–
	G	100 (100)	100 (100)		–
	C	0 (0)	0 (0)	–	

a Numbers in brackets denoted sample size.

## Discussion

The TPMT gene spans ∼34 kb and consists of 10 exons located on chromosome 6q22.3, encoding a monomeric 28 kDa protein of 245 amino acids.^35^ Genetic polymorphisms in TPMT contribute to significant interindividual variability in drug metabolism. Several nonsynonymous TPMT variants, including TPMT2 (A80P), have been associated with reduced or absent enzymatic activity and an increased risk of drug-related toxicity.^36^ Ethnic variation in TPMT polymorphisms has been well documented: the TPMT2 allele is rare or absent in Asian populations, including South Asians, but is more frequent among Caucasians and African-Americans.^10,11,11,12,12–37^ In silico studies have predicted that several variants, including A80P, disrupt structural stability and enzymatic function.^38^ Given the pharmacogenetic importance of TPMT and the known clinical relevance of its A80P variant (TPMT2), we aimed to characterize the mutation’s structural and functional impact through molecular modeling and assess its prevalence in a North Indian AML cohort. To this end, we employed all-atom molecular dynamics (MD) simulations, principal component analysis, molecular docking, steered MD, and umbrella sampling to comprehensively assess the structural and functional effects of the A80P variant.

The present study was conducted to determine the effect of the TPMT A80P mutation (TPMT2) on the structural dynamics, conformation, and binding of the TPMT cofactor by using classical and enhanced MD simulation techniques. Together, the frequency of the TPMT2 variant was also monitored by performing genotyping PCR on 50 AML patient samples. The structure of TPMT was retrieved from the PDB, and missing residues at the beginning of the N-terminal were modeled through Modeler. Simultaneously, the tertiary structure of the A80P MT was constructed, and both WT and MT models were energy-minimized following structure validation prior to making them available for MD simulation. Henceafter, MD simulation was conducted, and the gmx rmsd, gmx rmsf, and gmx gyrate modules of the GROMACS suite were utilized to explore the structural and dynamic differences caused by the A80P mutation. It was found that the A80P MT showed more deviation and higher compactness of the TPMT protein. Further, the flexibility of the TPMT structure was more pronounced in the mutant protein, thus demonstrating that the flexibility of TPMT is altered upon the A80P mutation. The altered flexibility was mainly accompanied by the residues lying on the turn regions that connect the β-sheets. The obtained results indicated that the A80P mutation disrupts the stability and dynamics of the TPMT protein.

Time-dependent structural-functional relationship of TPMT upon point mutations are achieved through measuring SASA, secondary structure components that make up up the overall tertiary structure, and the number of H-bonds formed within the protein and with the solvent. This task is achieved by using the gmx sasa, do_dssp tool, and gmx hbond modules of GROMACS. SASA is further decomposed into hydrophilic and hydrophobic SASA to characterize the driving forces behind protein stability, folding, and intermolecular interactions. During SASA analysis, it was found that A80P MT showed larger total and hydrophobic SASAs and a greater involvement of hydrophobic residues in intermolecular interactions. This suggested that the point mutation increases the surface area of the protein accessible to the solvent and disrupts protein folding. The A80P mutation is located at α-helix3, which distorts the helical arrangement of the protein and increases the solvent exposure of adjacent residues, as also reported previously.^13^ Similarly, an alteration in secondary structures was found in the A80P mutant, signifying that the mutation leads to an unstable protein structure. H-bond results suggested that the mutation decreases protein–protein H-bonds while increasing protein–solvent H-bonds, implying that protein stability is disrupted upon the A80P mutation. Collective motions of the TPMT protein are studied by principal component analysis, and it was found that A80P MT exhibited larger motions compared to WT, suggesting that the mutation leads to conformational instability.

Molecular interaction between the receptor (TPMT) and ligand (SAM) is assessed by means of docking. Prior to final docking, the reliability of computational docking is checked by comparing the experimental binding interaction obtained from the PDB, revealing a high degree of overlap in key interacting residues. Docking results suggested that the WT-ligand complex showed a slightly lower docking score than the A80P-ligand complex, indicating that the SAM ligand has a higher binding affinity toward the WT protein as compared to the MT protein. However, docking is considered a reliable approach for studying the binding interactions of macromolecules. Sometimes, the docking algorithm fails to predict the accurate binding affinity in order to save computational time by considering the receptor as a rigid molecule.^39^ The receptor plays an important role during ligand binding by acquiring the appropriate conformation for achieving the best binding mode. Therefore, binding stability and rescoring of binding energy are computed through analysis of the MD simulation trajectories of both docking complexes (WT- and A80P-SAM).

MD simulations for both WT and A80P MT complexes are stabilized, and trajectories are well equilibrated. Both RMSD and Rg for WT and A80P MT showed steady and consistent behaviors, with a sudden jump observed in the WT complex RMSD, probably due to local structural mobilization. Furthermore, some fluctuations are seen in the A80P MT complex, suggesting that the MT affected the overall flexibility of the A80P-SAM complex. The flexibility is mostly restricted to the N-terminal region of the protein. However, the flexibility in both the apo and complex forms of WT remains unaffected. It has been previously found that SAM increases the stability of TPMT, and a change in a single amino acid has a dramatic effect on the stability and function of the protein.^40^ Also, a structural dynamics study of the TMPT*23 variant, having the mutation of Alanine to Glycine at position 167 (A167G), has shown a decrease and increase in the flexibility of the region around the thiopurine and SAM binding site, respectively.^41^ The stabilized trajectories are used for calculating the binding free energy of SAM toward WT and A80P MT proteins. In total, binding free energies are calculated by summing the various binding energy forces (van der Waals, electrostatic, polar solvation, and SASA energies), and results suggested that the WT-SAM complex displayed lower binding free energy as compared to the A80P MT complex. This demonstrated that A80P MT lowers the binding affinity of SAM toward the TPMT protein, which ultimately affects the function of the protein. To further calculate the binding energy and trace the path of ligand unbinding, steered MD (SMD) simulation is performed, followed by umbrella sampling simulations. SMD is a technique that pulls the ligand from the binding site of the protein. It assesses the force profiles, which study the ligand binding strength, and results suggested that higher force is required to pull the ligand from the active site of WT as compared to A80P MT. Furthermore, PMF profiles of ligand binding with both WT and A80P MT are computed and found that a higher energy barrier for ligand unbinding to WT as compared to MT existed, suggesting low binding affinity with the MT protein. We further integrated and correlated the scoring values obtained from molecular docking, MM/PBSA calculations, and potential of mean force (PMF) analysis derived from umbrella sampling. Although the absolute binding energy values varied among docking, MD-based free energy estimations, and umbrella sampling approaches, a consistent trend was observed across all methods. Notably, the WT complex demonstrated higher binding affinity compared to the A80P mutant. This concordance across independent computational approaches supports the conclusion that the A80P point mutation compromises SAM binding, which may ultimately impair the functional activity of the TPMT protein. It was found that residue 80 is positioned on α-helix 3 of the protein, and substitution of Alanine to Proline disrupts the H-bond with Met76, which ultimately distorts the helical structure.^13^ Since this position is near the SAM binding site, the mutation leads to changes in the local packaging around the active site, resulting in affected SAM binding.

The study was further extended to determine the prevalence of TPMT2 polymorphism in AML patients and healthy controls in the North Indian population. We specifically selected AML patients because TPMT polymorphisms have been associated with altered response and toxicity to thiopurine drugs, which are often used in hematological malignancies, including protocols for leukemia treatment and supportive care.^9^ Thus, understanding the TPMT2 prevalence in AML patients could inform future personalized therapy and pharmacogenetic screening strategies. PCR-based genotyping of GG, GC, and CC genotypes was performed, and results revealed that the TPMT2 allele is absent in the above population. It was found in previous studies that the TPMT2 variant is also absent in other parts of the Indian population .^6,42^ It is clear from the current study that South-West Asia, particularly the Indian population, does not show the TPMT2 variant and exhibits normal activity of TPMT.

## Conclusions

In this study, we comprehensively investigated the structural and functional impact of the TPMT2 (A80P) variant using molecular dynamics simulations, docking, and enhanced sampling techniques. Our results demonstrate that the A80P substitution compromises TPMT stability by increasing backbone flexibility, expanding the solvent-exposed surface area, and disrupting intraprotein hydrogen bonding and β-sheet content. These changes weaken the overall structural integrity of the enzyme. Further, molecular docking and umbrella sampling simulations revealed that the A80P mutation significantly reduces the binding affinity for S-adenosylmethionine (SAM), with a markedly lower binding free energy and a reduced energy barrier for ligand dissociation (∼90 kJ/mol vs >120 kJ/mol in WT). These findings suggest impaired enzymatic function, potentially altering thiopurine drug metabolism in individuals carrying this variant.

We also screened 50 AML patients and 50 healthy controls from a North Indian cohort using allele-specific PCR. The TPMT2 variant was absent in all individuals, consistent with prior studies reporting its rarity in South Asian populations. While routine clinical testing for this variant may be unnecessary in this region, our structural analysis highlights its potential functional consequences when it is present. However, further investigation with larger and more diverse cohorts is required to more accurately evaluate the association of this variant with AML risk. Nevertheless, our findings provide mechanistic insights into the functional consequences of the TPMT point mutation and contribute to the growing body of evidence regarding the prevalence and clinical relevance of TPMT2 variants. These observations may have important implications for TPMT-guided pharmacogenomic strategies.

## Supplementary Material



## Data Availability

All data sets generated for this study are included in the article/Supporting Information.

## Notes

Structural modeling, docking, and MD simulations analyses of TPMT WT and A80P variant (Figures S1–S9) (PDF)
