# Supplementary material for: Structural and Functional Disruption of Thiopurine S‑Methyltransferase by the A80P Variant: A Simulation and Genotyping Study
Source: ACS Omega. 2026 Jun 17;11(25):36468–77. doi: 10.1021/acsomega.5c08089 (PMC13325393; doi:10.1021/acsomega.5c08089)
Supplement: Supplementary file 1 [file ao5c08089_si_001.pdf]

Supplementary information

Structural and Functional Disruption of Thiopurine S-methyltransferase by the A80P Variant: A Simulation and Genotyping Study

Kaishiv Joshi[1], Rahul Kumar[2], Rakesh Kumar[3*], Satbir Kaur[1] and Sebastian Kmiecik[3]

[1]Department of Human Genetics, Punjabi University, Patiala-147002, India

[2]Dr. B.R.A. Institute Rotary Cancer Hospital, All India Institute of Medical Sciences, New Delhi-110029, India

[3]Biological and Chemical Research Center, Faculty of Chemistry, University of Warsaw, Warsaw 02-089, Poland

***Corresponding author:** Rakesh Kumar

Biological and Chemical Research Center, Faculty of Chemistry, University of Warsaw, Warsaw 02-089, Poland

Email: rakeshkapoor.jnu@gmail.com; r.kumar2@uw.edu.pl

Supplementary figures : 9

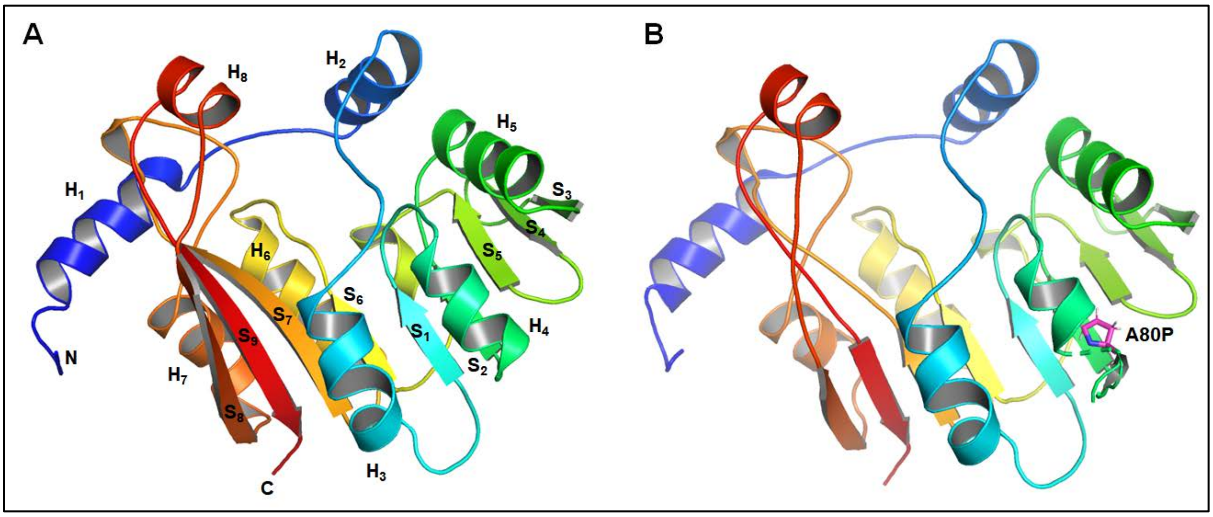

Figure S1. (A) Tertiary structure of TPMT modelled through modeller. Helices and sheets were represented as H and S, respectively. **(B)** Tertiary structure of TPMT showed the position of A80P mutation (magenta stick). Structure was shown as publication cartoon mode in PyMOL.

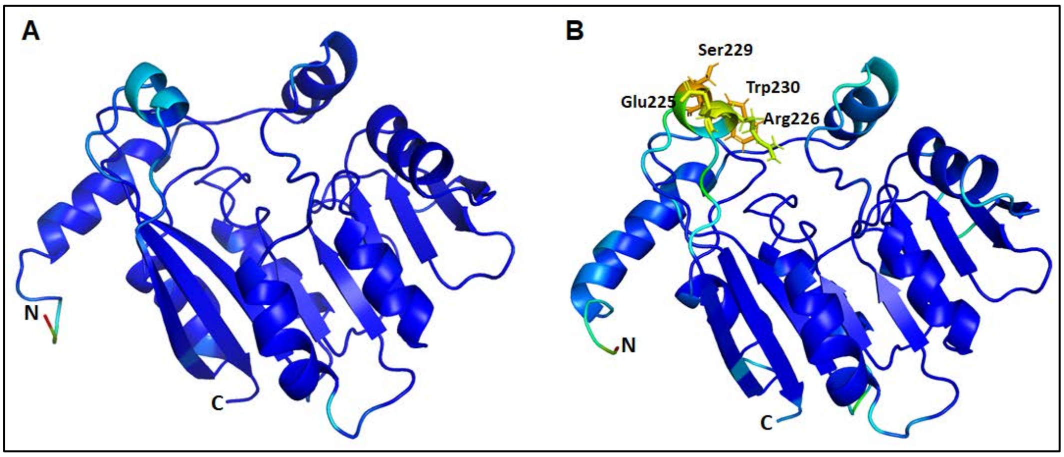

Figure S2. Root mean square fluctuation analysis (A) Wildtype and (B) A80P. Highly fluctuated residues are denoted as stick and labelled in three code letter residues.

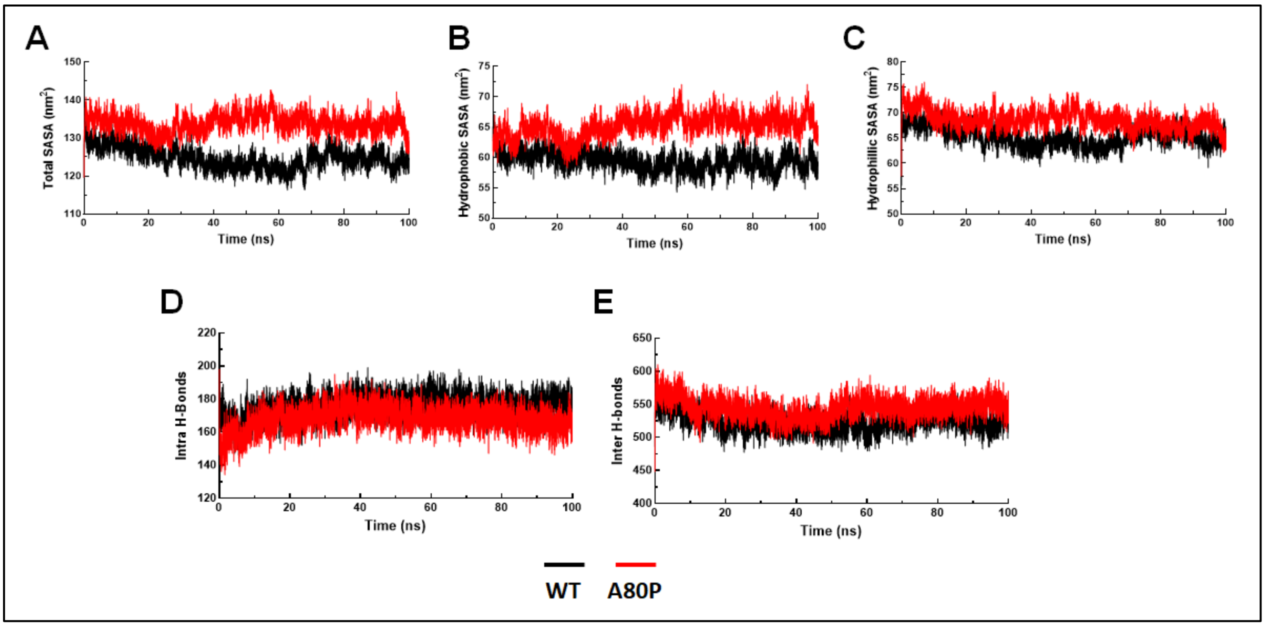

Figure S3. (A) Total SASA, (B) Hydrophobic SASA, (C) Hydrophilic SASA (D) Intra hydrogen bonds and (E) Inter hydrogen bonds. WT and A80P are shown in black and red colour lines, respectively.

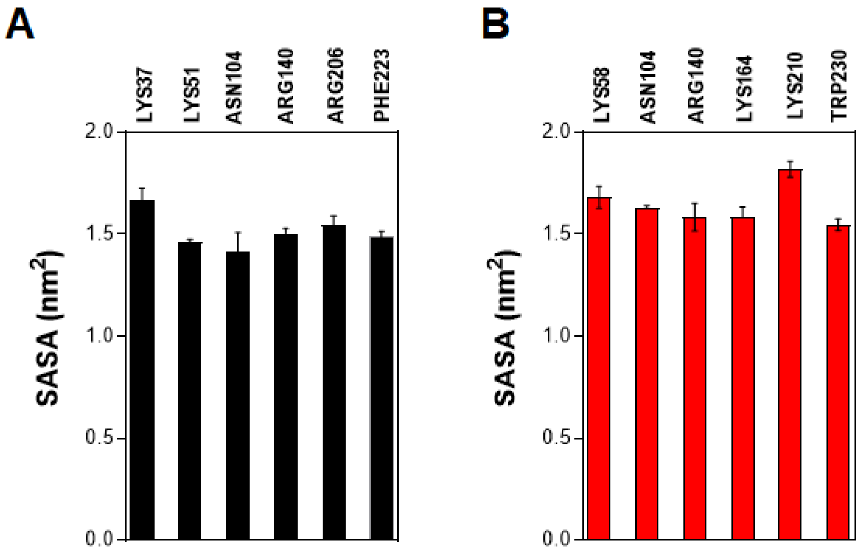

Figure S4. Residues level SASA in (A) WT and (B) A80P.

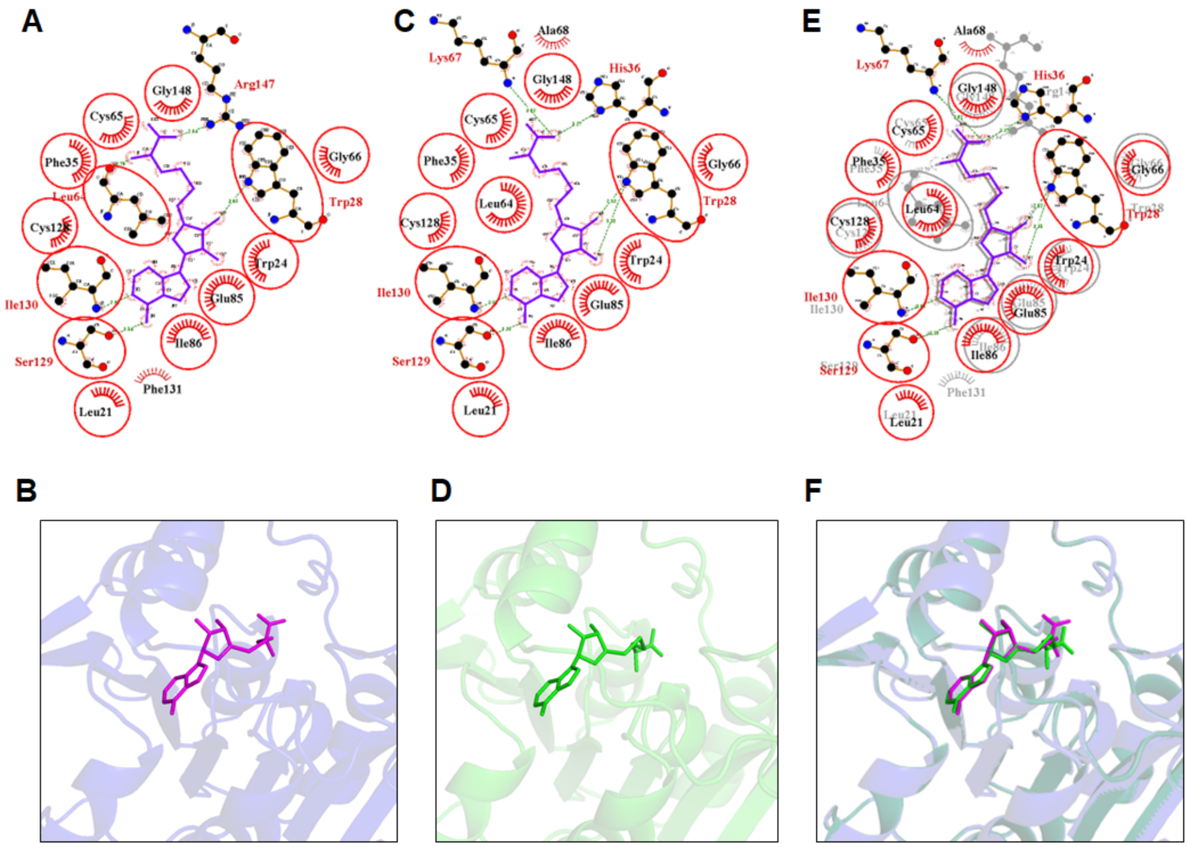

Figure S5. 2D and 3D plots of (A and B) Binding pose from docking, (C and D) Binding pose from PDB structure and (E and F) Superposition of docking and PDB complexes. 3D receptor and ligand are depicted in cartoon and stick modes with uniform colour.

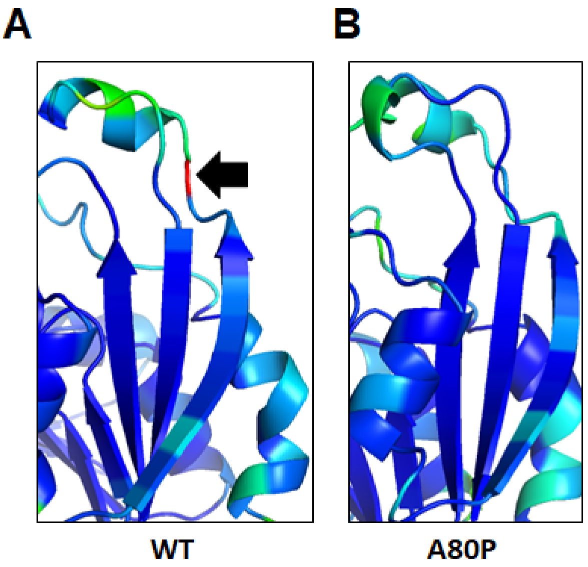

Figure S6. Mobility assessment of (A) WT and (B) A80P. Black arrow denoted highly mobile region.

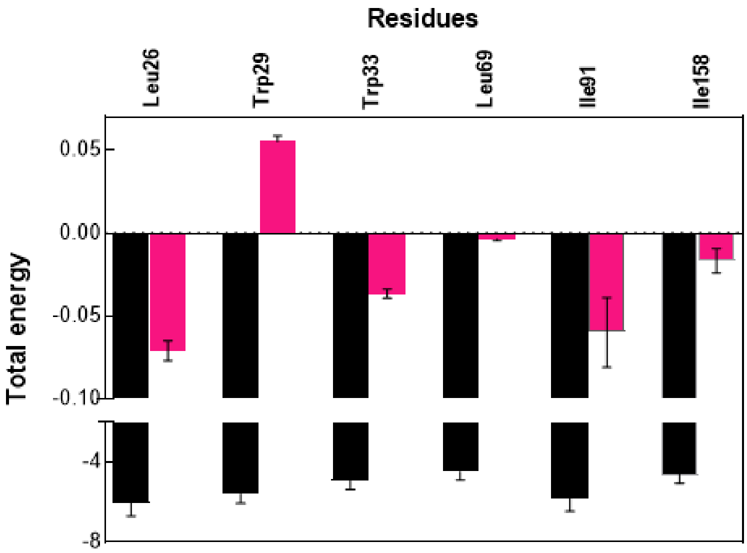

Figure S7. Free energy distribution on residues basis. WT and A80P are shown in black and magenta, respectively.

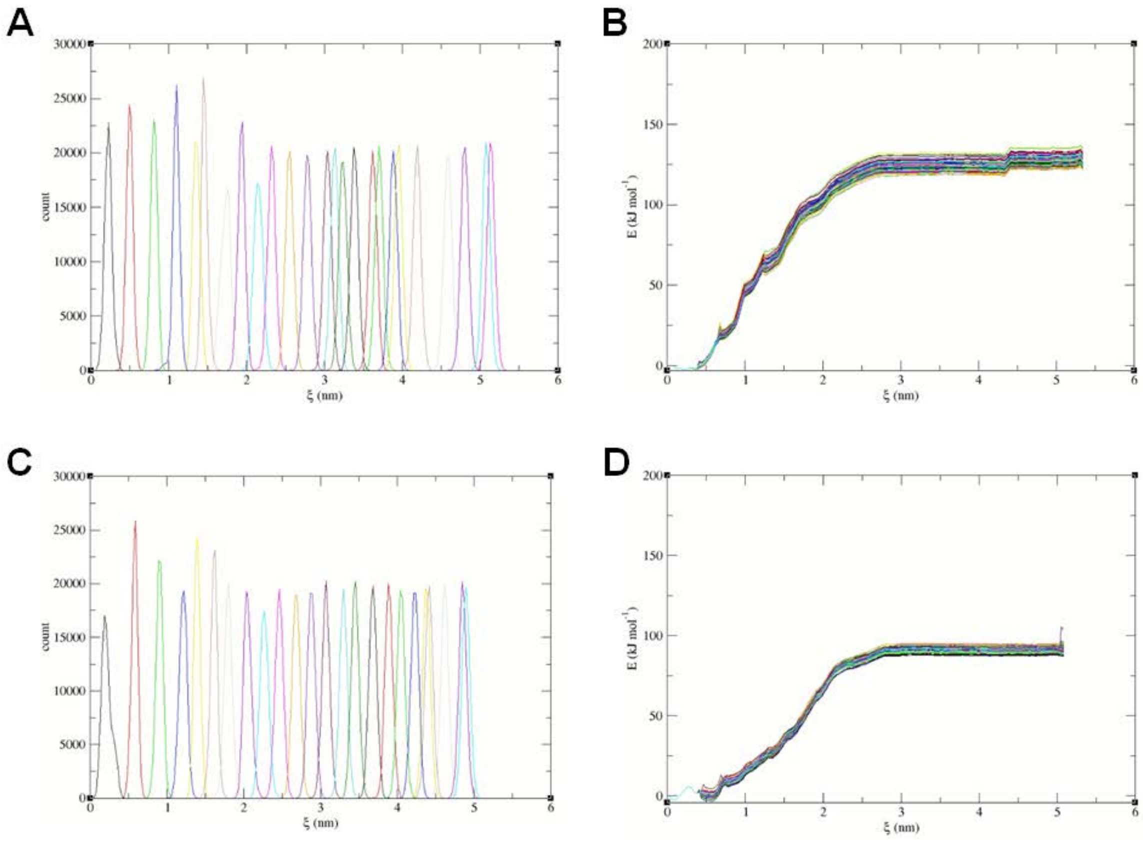

Figure S8. (A) Umbrella histogram and (B) PMF bootstrap graph of WT, (C) Umbrella histogram and (D) PMF bootstrap graph of A80P.

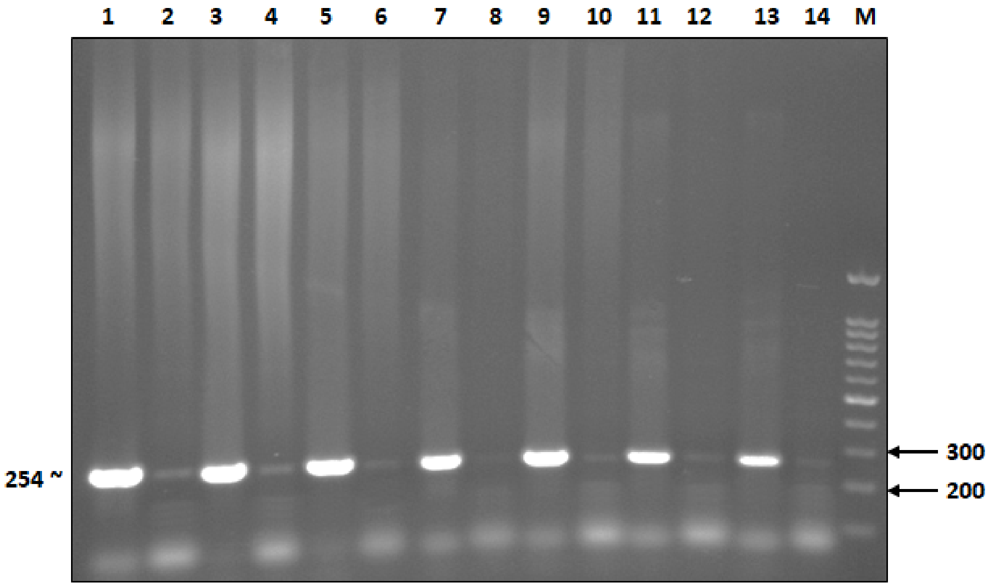

Figure S9. Agarose gel picture showing PCR product (~254bp) from represented samples of AML. M denoted DNA marker
